# PAK3 promotes the metastasis of hepatocellular carcinoma by regulating EMT process

**DOI:** 10.7150/jca.61918

**Published:** 2022-01-01

**Authors:** Zhi Gao, Mengya Zhong, Zhijian Ye, Zhengxin Wu, Yubo Xiong, Jinsong Ma, Huiyu Chen, Yuekun Zhu, Yan Yang, Yongxiang Zhao, Zhiyong Zhang

**Affiliations:** 1 National Center for International Research of Bio-targeting Theranostics, Guangxi Key Laboratory of Bio-targeting Theranostics, Collaborative Innovation Center for Targeting Tumor Diagnosis and Therapy, Guangxi Talent Highland of Bio-targeting Theranostics, Guangxi Medical University, China.; 2 Department of Gastrointestinal Surgery, Zhongshan Hospital of Xiamen University, Xiamen, Fujian, China.; 3 School of Medicine, Xiamen University, Xiamen, Fujian, China.; 4 School of Medicine, Guangxi University, Nanning, Guangxi, China.; 5 Department of General Surgery, The First Affiliated Hospital of Harbin Medical University, Harbin, China.; 6 Medical Center, Duke University, Durham, NC.; 7 Department of Surgery, Robert-Wood-Johnson Medical School University Hospital, Rutgers University, The State University of New Jersey, New Brunswick, NJ.

**Keywords:** PAK3, HCC, EMT, smad

## Abstract

**Purpose:** Hepatocellular carcinoma (HCC) is one of the most common malignant tumors. The malignant biological behavior of HCC is closely related to epithelial-mesenchymal transition (EMT), and EMT plays an important role in the progression, migration and metastasis of HCC. P21-activated kinase 3 (PAK3) is a serine/threonine protein kinase, and PAK3 affects the EMT, proliferation, metastasis and invasion of HCC.

**Methods:** In this study, the relationship between PAK3 and HCC was first analyzed by bioinformatics, and then, the expression of PAK3 in clinical samples was detected by immunohistochemistry (IHC), quantitative real-time PCR (qRT-PCR) and Western blotting. Subsequently, the expression of PAK3 was further confirmed in HCC cells. In addition, after the overexpression or knockdown of PAK3 in cells, the proliferation, migration and invasion abilities of these cells were assessed by Cell Counting Kit-8 (CCK-8), wound healing and Transwell assays, and the results were confirmed *in vivo* experiments in mice. In addition, we also verified that PAK3 affected the EMT and EMT-related pathway of HCC through qRT-PCR, Western blotting and immunofluorescence experiments.

**Results:** Through database analysis, we found that PAK3 was highly expressed in HCC patients and was positively correlated with tumor stage and grade, suggesting that PAK3 expression was closely related to HCC occurrence and development. We subsequently confirmed that PAK3 was overexpressed in HCC clinical samples and HCC cell lines and that PAK3 promoted the proliferation, migration and invasion of HCC cells *in vitro*. Finally, we found that PAK3 regulated EMT-related molecule expression and EMT-related TGF-β/smad signaling pathway.

**Conclusion:** High expression of PAK3 enhances the invasion of HCC and regulates EMT, suggesting that PAK3 may be a potential target for the treatment of HCC.

## Introduction

Hepatocellular carcinoma (HCC) is the third most common cause of cancer-related death, and its incidence is expected to increase worldwide 1. Due to the lack of clear symptoms and specific biomarkers, most HCC patients are diagnosed in the late stage of disease, and patients with advanced HCC are generally not eligible for surgical treatment and have short survival periods, usually only 6 months 2. Therefore, the treatment of HCC is still a difficult problem. It is of great significance to understand the mechanisms underlying HCC metastasis and to identify new targets for the early diagnosis and treatment of HCC.

P21-activated kinases (PAKs) are serine/threonine protein kinases that are stimulated by the GTPases Cdc42 and Rac and bind to their activated forms under certain conditions. PAK family members are overexpressed or overactivated in many tumors 3. PAKs are divided into two groups based on structural similarity. Group I PAKs include PAK1 through 3 4. PAK3 is abundantly expressed in the central nervous system (CNS) and is specifically implicated in neuronal plasticity and spinogenesis. PAK3 also regulates cell cycle progression, neuronal migration and apoptosis 5. Recent research has shown that aberrant expression and activation of PAKs occur during tumor pathogenesis, causing PAKs to be considered tumor enhancer proteins. PAK3 acts as an oncogene to promote the progression of gastric cancer and can be regulated by Circ_0000190 6. PAK3 can also promote pancreatic cancer stem cell phenotypes by activating the Akt-GSK3β-β-catenin signaling pathway 7. However, the association between PAK3 and HCC has not been elucidated.

Epithelial-mesenchymal transition (EMT), which is a cellular reprogramming process by which epithelial cells dramatically alter their shape, is essential for tumor metastasis and plays a key role in the progression of HCC 8. During HCC progression, cells lose cell-to-cell contact due to the loss of E-cadherin and acquire migration abilities to spread to surrounding or distant tissues, thus promoting the invasion and metastasis of cancer. This process plays a crucial role in the early stages of metastasis 9. Therefore, inhibiting EMT is an essential means by which to suppress tumor metastasis. Increased TGF-β signaling is key for enhancing EMT during cancer progression and metastasis. Smad proteins are downstream targets of TGF-β signaling, and these proteins play important roles in regulating transcription and controlling cell proliferation and differentiation. Phosphorylation of Smads can promote the transition from antitumor phenotypes to tumorigenic phenotypes.

In this study, we determined the role of PAK3 in HCC and the correlation between PAK3 expression and EMT in HCC. Through biochemical analysis and clinical sample assessment, we found that PAK3 was highly expressed in HCC, and we overexpressed or knocked down PAK3 in HCC cells. Functional experiments showed that PAK3 was closely related to tumor proliferation, metastasis and invasion. Subsequently, transcription factors and markers related to EMT were found to be altered by the overexpression of PAK3 in HCC cells. Moreover, the levels of p-Smad2 and p-Smad3, components of the TGFβ/Smad pathway, which is important for EMT, were also increased. In summary, we showed that PAK3 is an oncogene in HCC. PAK3 promoted EMT to regulate the progression of HCC. In this process, TGF-β/Smad signaling was activated and involved in the entire regulatory process.

## Material and methods

### HCC tissue collection

In this study, HCC tissue samples and corresponding non-tumor samples were collected from HCC patients at Harbin Medical University. This study was approved by the ethic committee at Harbin Medical University. Informed consent was obtained from each patient. The diagnosis was confirmed as HCC based on World Health Organization criteria. All patients were admitted to hospital within the 3 months. No antitumor therapy, including percutaneous ablation, radiotherapy, or chemoembolization, was performed before the surgery. The patient did not have other serious diseases. Patients with tumor history should be excluded. For patient information, see supplementary Table 1. All tumor tissues used for RNA isolation, Western blotting and immunohistochemistry (IHC) were confirmed by a pathologist.

### Lentiviral transduction and cell transfection

Lentiviral PAK3 shRNA plasmid, PAK3 expressing vector and their negative control vector were obtained from Genecopoeia (Guangzhou, China). For lentivirus package, constructs and packaging plasmids were transfected into 293T cells using Lentiviral Packaging Kit (Yeasen, China). The media containing the lentivirus were collected 72 h after transfection, and purified virus particles were used to infect HCC cell lines. The information of shPAK3 is: GGCACGATTACTCCAAACTTC. qRT-PCR and western blot were performed to confirm the efficacy of viral transduction and cell transfection.

### Cell culture

The human HCC cell lines HepG2, Hep3B, Huh7, and HCCLM3 were purchased from Zhong Qiao Xin Zhou Biotechnology (ShangHai, China). The HepG2, Huh7, and HCCLM3 cells were cultured in Dulbecco's modified Eagle medium (DMEM, Gibco, USA), and the Hep3B cells were cultured in minimum essential medium (MEM, Gibco, USA). All the media were supplemented with 10% fetal bovine serum (FBS, A0500-3011, Cegrogen Biotech, Germany) and 1% penicillin-streptomycin (Meilunbio, China), and the cells were cultured at 37°C in a 5% CO_2_ air atmosphere.

### Quantitative real-time PCR

Quantitative real-time PCR (qRT-PCR) was performed in 96-well plates using the TransStart Top Green qPCR SuperMix (TransGen Biotech, Beijing, China) according to the instructions. qRT-PCR was carried out on a Bio-Rad Biosystems 7500 instrument with SYBR Green (Bio-Rad, Hercules, CA). Total RNA was extracted from cells using the TransZol reagent (Thermo Fisher, USA). Then, the RNA (1 μg) was reverse-transcribed into cDNA using the All-in-One First-Strand cDNA Synthesis SuperMix kit (TransGen Biotech, Beijing, China). The relative expression of the target genes was calculated with the 2^-ΔΔCt^ method based on their Ct values, and 18S rRNA was used as the internal control and its sequence-specific primers were: 18S forward, CTTAGAGGGACAAGTGGCG, reverse, ACGCTGAGCCAGTCAGTG; PAK3 forward, CAACCGGGATTCTTCAGCACT, reverse, CACATGAATCGTATGCTCAAAGTCTG; Snail forward, CAGCTATTTCAGCCTCCTGTT, reverse, CCGACAAGTGACAGCCATTA; Zeb1 forward, GGCTCCTATAGCTCACACATAAG, reverse, TGCTGAAAGAGACGGTGAAG; Twist1 forward, AGACTCTGGAGCTGGATAACT, reverse, GCCTGTCTCGCTTTCTCTTT.

### Western blotting and antibodies

To prepare the protein, cells were lysed with radioimmunoprecipitation assay (RIPA) lysis buffer (Meilunbio, China) containing a 50X phosphatase inhibitor cocktail, 100X phenylmethyl sulfonyl fluoride (PMSF) and a 100X protease inhibitor cocktail. The concentrations of the protein samples were measured with a Bicinchoninic Acid (BCA) Protein Assay Reagent kit (Thermo Fisher, USA). Equal amounts of proteins were denatured in 5X loading buffer. Then, the cellular proteins were loaded into 12% or 8% SDS-PAGE gels. After electrophoresis, the proteins were transferred to PVDF membranes (Millipore, MA). The membranes were blocked with 5% nonfat milk and incubated with primary antibodies overnight at 4°C. After primary antibody incubation, the PVDF membranes were washed three times for 30 min in TBST buffer (0.1% Tween in PBS) and then incubated with horseradish peroxidase-conjugated secondary antibodies for 1 h at room temperature. Finally, the membranes were washed three times, and the proteins were visualized using enhanced chemiluminescence (ECL) on a Tanon 5200 instrument.

### Cell Counting Kit-8 assay

Cell growth was examined with Cell Counting Kit (CCK)-8 (Dojindo) according to the manufacturer's instructions. Cells were transfected and cultured for 12, 24, 48 and 72 h at a density of 5000 cells per well in 96-well plates. Then, 10 μL of CCK-8 solution was added to each well, and the cells were cultured for another 2 h at 37°C. The absorbance was read at 450 nm using a microplate reader.

### Wound healing assay

Transfected Huh7 cells were seeded into 6-well plates. Once the cells reached 80% confluence, the cell monolayer was wounded by scraping with a pipette tip. Then, the cells were cultured in serum-free media. The wound size was monitored at 0 h and 24 h by a Leica DM4B microscope (Leica, Germany).

### Transwell migration and invasion assays

Polycarbonate filters with 8-μm pores (Corning Costar) combined with 24‐well culture plates were used for migration (noncoated) and invasion (Matrigel‐coated) assays. Cells (8×10^5^ cells/mL) were collected and resuspended in 100 µL serum‐free DMEM. Then, the cells were seeded on each polycarbonate filter in the 24‐well plates, and the bottom chambers contained 600 µL 20% FBS‐DMEM. After incubation at 37°C for 24 h, the cells were fixed in 4% paraformaldehyde and stained for 30 min in a 0.1% crystal violet solution in PBS. The number of cells on the underside of each insert was determined using light microscopy (Olympus, Japan). Five randomly selected fields were counted per insert.

### Immunofluorescence

HepG2 and Huh7 cells were plated on coverslips in 6-well plates at a density of 50000 cells per well. After the cells adhered to the well, they were fixed with 4% paraformaldehyde and then permeabilized with 0.2% Triton X-100 for 20 min on ice. After washing with phosphate-buffered saline (PBS) 3 times for 10 min, the cells were blocked with 2% bovine serum albumin (BSA) for 1 h and then incubated overnight at 4°C with primary antibodies. The cells were washed with PBS and incubated with Alexa Fluor 488- or 594-conjugated secondary antibodies for 1 h at room temperature; the nuclei were stained with Hoechst 33342 (Beyotime, Shanghai, China), and images were captured with a CLSM fluorescence microscope (Zeiss LSM 880+Airyscan, Carl Zeiss AG, Germany).

### HCC metastasis model

All male BALB/c nude mice (4-6 weeks of age) were used for tail vein injection experiments to evaluate the metastatic ability of different HCC cells *in vivo*. Hep3B-pLV-control (1×10^6^) and Hep3B-pLV-PAK3 cells (1×10^6^) were injected into the tail veins of nude mice. At indicated times, the lungs of nude mice were removed and embedded in paraffin for H&E staining. Animal protocols were approved by the Institutional Animal Care and Ethics Committee of Xiamen University, Review Number: XMULAC 20200080.

### Tissue arrays and immunohistochemical (IHC) staining

HCC tissue microarray was purchased from US Biomax Company. The tissue microarray was stained for PAK3 (abcam, ab40808) expression. Immunochromogenic kits and DAB staining solution (Fujian Maixin) were used for IHC staining.We carried out the experiment according to the instructions. IHC staining was scored by the scale: 0, 1+, 2+, and 3+, representing no staining, weak, moderate, and strong staining, respectively. The indicated protein levels were defined by H-score; then, low and high expression patient groups were divided.

### Data collection and statistical analysis

The gene expression profile of 423 liver hepatocellular carcinoma (LIHC), including 50 pairs of tumor and adjacent normal tissue was obtained from the Cancer Genome Atlas (TCGA) (https://portal.gdc.cancer.gov/). The Student t-test was used to assess differences in variables between groups. The Kaplan-Meier analysis and log-rank test were used to explore the prognostic implication of PAK3 in TCGA LIHC cohort. The optimal cutoff value was identified using X-tile software. The prognostic value of PAK3 was further estimated by univariate and multivariate Cox proportional hazard model analysis. To explore the latent biological processes of PAK3, the LIHC cases were classified into four groups. The first and fourth quarter of samples were then subjected to gene set enrichment analysis (GSEA). All statistical analyses were performed using SPSS Statistical software (version 19.0) and R project (version 3.6.0). A two-side p-value less than 0.05 was considered as significant.

## Results

### PAK3 is associated with HCC which predicts poor prognosis

**Figure 1:**
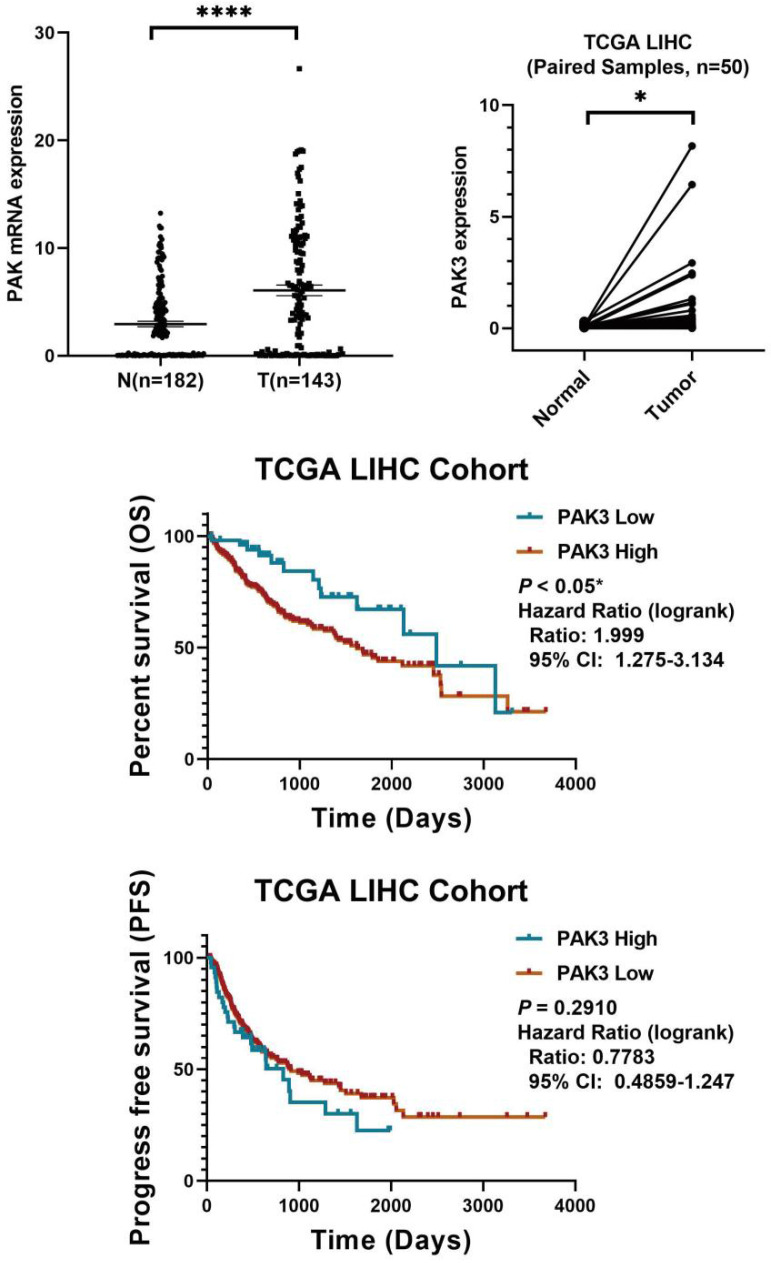
** PAK3 up-regulation is tightly associated with HCC malignancy and poor survival of patients. (a)** The GSE138485 data confirmed that PAK family members are highly expressed in HCC. **(b)** Relative expression of PAK3 in 50 HCC patients quantified by quantitative real-time PCR and compared with the adjacent normal samples. **(c and d)** Patients with HCC were stratified by the PAK3 level, OS and DFS were assessed by Kaplan-Meier analysis.*P < 0.05; **P < 0.01; ****P<0.0001.

To investigate the role of PAK3 in human HCC, we first analyzed the expression of PAK family in the GSE138485 data set of the Gene Expression Omnibus (GEO) database. The results revealed that the expression of PAKs was increased in the HCC samples in the GEO database (Figure 1a). Next, we checked the function of PAK3 in HCC in the Cancer Genome Atlas (TCGA) database and verified that the PAK3 mRNA expression level was significantly increased in HCC tissues compared with that of normal tissues (Figure 1b). Finally, we obtained clinical information from TCGA to evaluate the prognostic significance of PAK3 in HCC. Kaplan-Meier analysis indicated that HCC patients with higher expression of PAK3 presented shorter disease-free survival (DFS) and overall survival (OS) times (Figure 1c and 1d). These results indicated that PAKs are relevant to the prognosis of HCC.

In sum, we found that PAK3 plays a vital role in the tumorigenesis and development of HCC. Our results suggest that pak3 is highly expressed in hcc and that increased expression of PAK3 is associated with poor prognosis in HCC patients. These findings suggest that PAK3 might play a critical role in HCC and be a valuable biomarker of this disease.

### PAK3 is significantly overexpressed in HCC tissues and HCC cells

**Figure 2:**
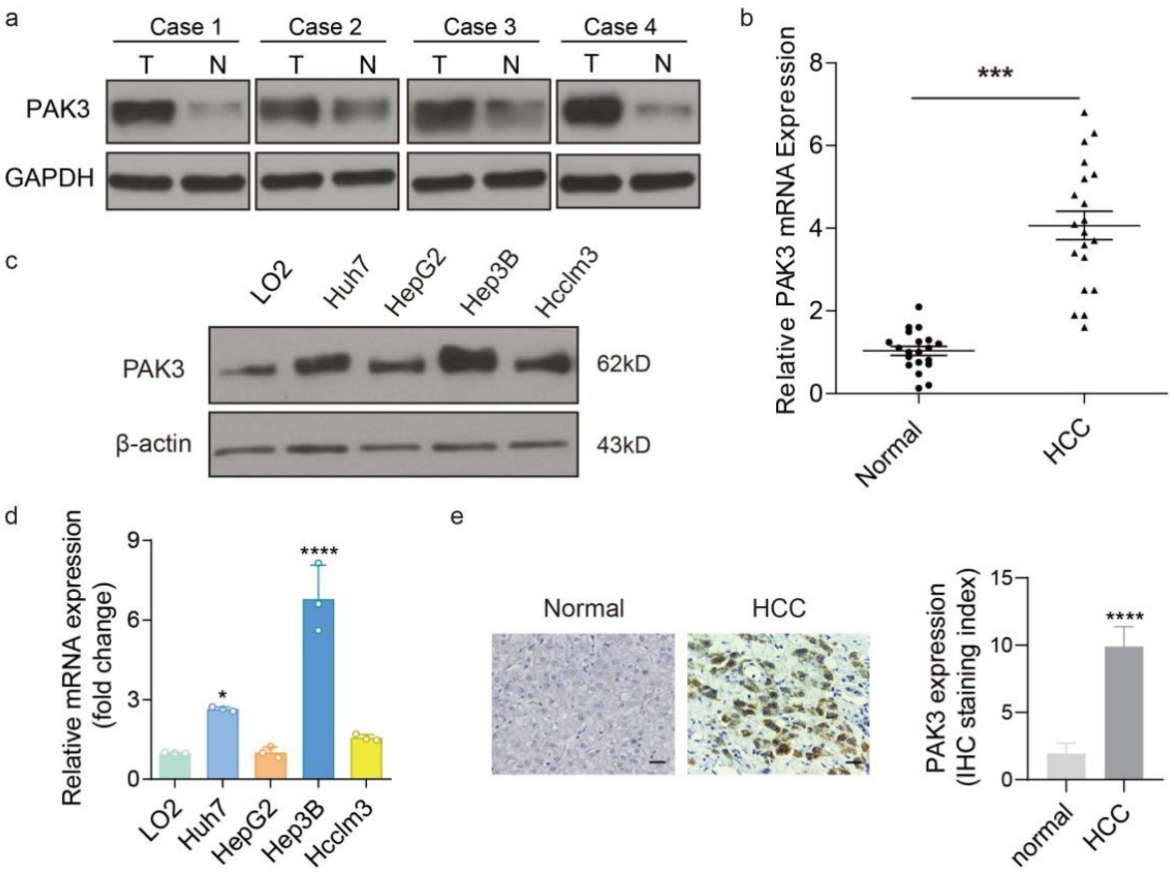
** PAK3 overexpressed in HCC tissues and cells. (a and b)** Protein and mRNA expression of PAK3 in HCC tissues and normal tissues. **(c and d)** Protein and mRNA expression of PAK3 in HCC cells compared with normal liver cell line. **(e)** IHC was used to detect PAK3 expression on HCC patients' tissue and healthy adjacent tissue, staining score as calculated. Scale bar= 50 µm, ***P < 0.001; ****P<0.0001.

To assess the expression of PAK3 in HCC, the protein and mRNA levels of PAK3 expression was examined in HCC tissues and adjacent tissues by western blotting and qRT-PCR. We found that the expression of PAK3 in HCC tissues was higher than that in normal tissues (Figure 2a and 2b). Additionally, the results of IHC also confirm this result (Figure 2c). Meanwhile, the mRNA and protein expression levels of PAK3 in HCC cell lines (HepG2, Hep3B, Huh7 and HCCLM3 cells) were also examined by qRT-PCR and Western blotting, and the results showed that PAK3 was also highly expressed in HCC cell lines (Figure 2d and 2e). Together, the results indicated that PAK3 was highly expressed in human HCC tissues and HCC cell lines.

### PAK3 promotes the proliferation, migration and invasion of HCC cells

**Figure 3:**
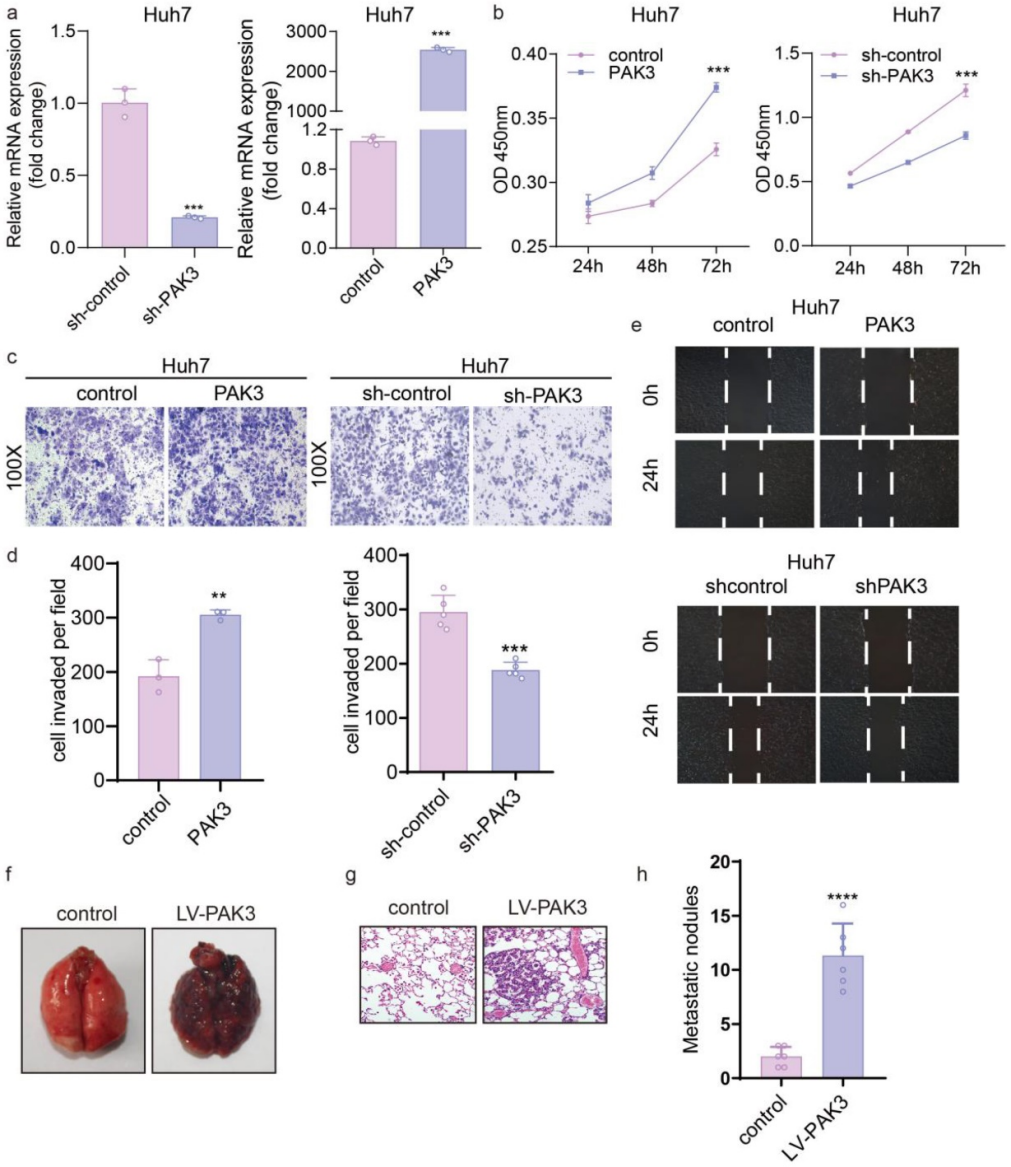
** PAK3 promotes the proliferation, migration, and invasion of liver cancer cells. (a)** PAK3 overexpression in Huh7 cells transfected with PAK3-pEZ-Lv206 and PAK3 inhibition in Huh7 cells transfected with LV-PAK3 shRNA were confirmed by qRT-PCR. **(b)** The CCK-8 assay was performed to evaluate the effects of PAK3 on Huh7 cell proliferation. **(c and d)** The effect of PAK3 on invasion was detected by invasion assay using PAK3 overexpression and knockdown Huh7 cells. **(e)** Migration assay was performed in the indicated cells. **(f and h)** Effect of PAK3 overexpression on lung metastasis model in mice. **(g)** HE was used to conform the malignancy of metastatic lung cancer. **P < 0.01; ***P < 0.001; ****P<0.0001.

To investigate the role of PAK3 in the proliferation of HCC cells, we overexpressed PAK3 in Huh7 cells using PAK3-pEZ-Lv206 and knocked down PAK3 in Huh7 cells using specific shRNA delivered by lentivirus (LV-PAK3 shRNA). PAK3-pEZ-Lv206 transfection significantly upregulated the expression of PAK3 in Huh7 cells, and LV-PAK3 shRNA markedly decreased the expression of PAK3 in Huh7 cells (Figure 3a). CCK-8 analysis showed that overexpression of PAK3 significantly promoted the proliferation of Huh7 cells, whereas the proliferation was significantly decreased when PAK3 was knocked down in Huh7 cells (P<0.05) (Figure 3b). This information indicated that PAK3 promoted the cell cycle progression of HCC cells, confirming that PAK3 promoted HCC cell proliferation.

We further evaluated the migration ability of HCC cells by wound healing assay and Transwell migration and invasion assays. The wound healing assay showed that compared with the control, overexpression of PAK3 in Huh7 cells promoted cell migration at the edge of the wound, whereas the migration distance of Huh7 cells transfected with LV-PAK3 shRNA was significantly shorter than that of control cells at 48 h after wounding (P<0.05) (Figure 3e). Consistently, the Transwell migration assays also revealed that PAK3 overexpression significantly increased the number of migrating cells, and PAK3 knockdown inhibited the motility of HCC cells (P<0.05). The Transwell invasion assays revealed that the numbers of invasive Huh7 cells were significantly increased after transfection with PAK3-pEZ-Lv206 (P<0.05). In contrast, the numbers of invasive Huh7 cells decreased after PAK3 expression was knocked down (P<0.05) (Figure 3c and 3d). These results demonstrated that PAK3 could enhance HCC cell migration and invasion.

*In vivo*, as clearly shown, cancer cells metastasized in the lungs of the PAK3 overexpression group, but no metastasis was observed in the control group, which indicates that overexpression of PAK3 significantly promotes tumor metastasis (Figure 3f-h).

### PAK3 enhances EMT through the TGF-β/smad pathway

**Figure 4:**
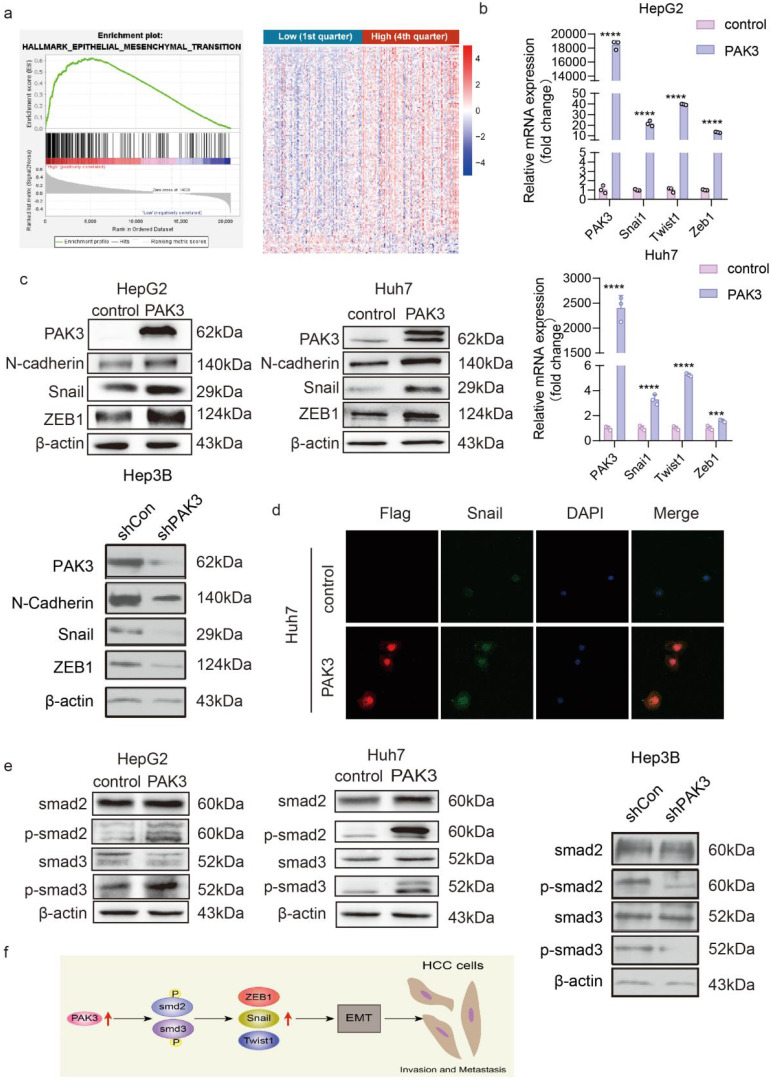
** PAK3 promotes the expression of EMT-related molecules through the TGFβ/Smad pathway. (a)** GSEA was used to found that PAK3 can promote EMT. **(b)** EMT-related transcription factor (Snail, Twist1, and ZEB1) expression in HepG2 and Huh7 cells was measured by qRT-PCR. **(c)** Western blotting was performed to detect EMT-related molecule (N-cadherin, Snail, and ZEB1) expression in HepG2, Huh7 and Hep3B cells. **(d)** Immunofluorescence analysis of PAK3 and Snail expression in Huh7 cells. **(e)** Western blotting assays show that PAK3 overexpression increases, while PAK3 knockdown decreases, Smad2 and Smad3 phosphorylation but does not affect total Smad2 or Smad3 expression. **(f)** Schematic depicting the function of PAK3 in HCC. ***P < 0.001; ****P<0.0001.

The GSEA of LIHC samples revealed that the hall marker gene sets of EMT were significantly enriched in patients with PAK3 high expression (Figure 4a, p < 0.05, normalized enrichment score = 1.540) (Figure 4a). We next explored the effect of PAK3 on the progression of EMT. The expression of EMT markers and transcription factors (N-cadherin, Snail, Twist1 and ZEB1) in Huh7 and HepG2 cells was examined by qRT-PCR and Western blotting. The results showed that overexpression of PAK3 in Huh7 and HepG2 cells increased the expression of N-cadherin, Twist1, Snail and ZEB1. Both qRT-PCR and Western blotting showed the same results (Figure 4b and 4c). These results indicated that PAK3 could regulate the expression of EMT-associated molecules to induce HCC initiation and proliferation. Further, it was also confirmed by immunofluorescence (Figure 4d).

Overexpression of PAK3 promoted EMT through the TGFβ/Smad2/3 pathway. The Western blot results confirmed this finding (Figure 4e). When PAK3 was overexpressed in HepG2 and Huh7 cells, the levels of p-Smad2 and p-Smad3 were also increased, while PAK3 knockdown had the opposite effects. Collectively, these findings suggest that targeting the PAK3 might be critical in the treatment of HCC (Figure 4f).

## Discussion

HCC accounts for approximately 90% of all primary liver cancer cases, with 850,000 new cases diagnosed each year. Surgical resection, liver transplantation, local radiotherapy, chemotherapeutic therapy, and combined therapy are the main treatment options for HCC. HCC rapidly develops and easily metastasizes, and advanced HCC is difficult to cure 10. Therefore, understanding the molecular biological mechanisms underlying the occurrence and development of HCC is helpful for early diagnosis and tumor classification as well as for identifying new therapeutic methods.

PAK3, a member of the PAK family, was first identified as a target of the Rho small GTP-binding protein family in 1995, and studies have established a role of PAK3 in nervous system development, such as in synaptic plasticity and spinal morphology 11, 12. However, there is little research on the role of PAK3 in the development of cancer. PAK1, which is a member of group I of the PAK family, is an important regulator of cell growth and metastatic phenotypes in cancer 13. PAK3, also a member of group I of the PAK family, has 90% homology with PAK1 in the kinase domain, and some active mutations of the PAK3 gene have been identified in some tumors through genome sequencing 14. These findings strongly suggest that PAK3 is involved in the etiology of tumors. At present, it has been confirmed that PAK3 may be involved in the development of thymus carcinoid, gastric cancer and pancreatic cancer, and PAK3 is considered a tumor enhancer that can promote the occurrence and development of such cancers. However, no relevant studies have been conducted about the relationship between PAK3 and HCC. In this paper, the function of PAK3 in HCC was studied at the molecular level.

In our study, we demonstrated that PAK3 was upregulated in HCC tissues and HCC cells. The proliferation ability of Huh7 HCC cells was enhanced after the overexpression of PAK3 compared with the control. When PAK3 was knocked down, Huh7 cell proliferation was reduced, suggesting that PAK3 promoted HCC proliferation. The Transwell assay results indicated that in Huh7 HCC cells, the invasion ability was increased after the overexpression of PAK3, and the invasion ability was decreased after the knockdown of PAK3. A cell wound healing assay also proved that PAK3 could promote the migration of Huh7 cells. These results suggest that PAK3 promotes the proliferation, migration and invasion of HCC cells.

EMT plays an extremely important role in the development of hepatoma. After the occurrence of EMT, the expression of epithelial marker genes, such as the E-cadherin and keratin genes, is decreased, while the expression of interstitial marker genes, such as the vimentin, fibroconnectin and N-cadherin genes, is increased. The occurrence of EMT can induce increased mobility of tumor cells and increased risk of lymph node and distant metastasis. The decrease in epithelial marker expression and the increase in stromal marker expression occur mainly through the cellular transcription process, which involves three transcription factor families, namely, the Snail, ZEB and Twist families 15. These transcription factors, once activated, can inhibit the expression of epithelial marker genes and simultaneously promote the expression of stromal genes. Snail, Twist, and ZEB all have the ability to independently regulate their targets and have been shown to have the potential to increase aggressiveness 16. The expression of these transcription factors can also be considered an indicator of high metastatic capacity.

The malignant biological behavior of liver cancer is closely related to the occurrence of EMT, while the mechanism underlying the relationship between PAK3 and EMT has not yet been reported. We have confirmed that PAK3 can promote the proliferation, migration and invasion of HCC through cellular functional experiments, and we think that these functions of PAK3 are caused by the promotion of EMT in HCC cells. Therefore, we detected the expression of EMT-related molecules after the overexpression of PAK3 in HCC cells. First, we detected the expression of EMT-related transcription factors in transfected liver cancer cells by qRT-PCR assay, and the results showed that the expression of EMT-related transcription factors increased with the increased expression of PAK3. Next, we detected the protein levels of EMT-related markers and transcription factors and found that after the overexpression of PAK3, the protein expression levels of N-cadherin, Snail and ZEB1 also increased. Immunofluorescence experiments also confirmed that the expression of PAK3 could promote the expression of Snail. It is suggested that high expression of PAK3 promotes HCC invasion and metastasis. These results also further support the conclusion that PAK3 expression is associated with distant tumor metastasis in HCC patients.

To understand the mechanisms by which PAK3 affects tumor formation, invasion, and metastasis as well as EMT in HCC cells, we next examined molecules involved in the relevant pathways. Multiple lines of evidence suggest that an increase in the TGF-β signaling pathway is key for enhancing EMT during cancer progression and metastasis. There are two types of TGF-β signaling pathways: the typical Smad-dependent pathway and the atypical Smad-independent pathway. TGF-β mediates a range of activities related to tumor progression through one or both pathways 17. Smad proteins are downstream targets of TGF-β signaling, and these proteins play important roles in regulating transcription and controlling cell proliferation and differentiation. The phosphorylation of Smads can promote the transition from antitumor phenotypes to tumorigenic phenotypes 18. We detected the expression of the important molecules Smad2, Smad3 and their phosphorylated forms. When PAK3 was overexpressed in Huh7 and HepG2 hepatoma cells, the levels of p-Smad2 and p-Smad3 were significantly increased, while the levels of total Smad2 and Smad3 were almost unchanged, suggesting that PAK3 may act through the Smad-dependent TGF-β pathway to promote EMT in hepatoma cells.

In summary, this study provides evidence that PAK3 is an oncogene in HCC and contributes to EMT in HCC by regulating Smad2 and Smad3, suggesting that PAK3 might be a potential biomarker and attractive therapeutic target for HCC.

## Supplementary Material

Supplementary table.
