# Supplementary material for: PAK3 promotes the metastasis of hepatocellular carcinoma by regulating EMT process
Source: J Cancer. 2022 Jan 1;13(1):153–61. doi: 10.7150/jca.61918 (PMC8692680; doi:10.7150/jca.61918)
Supplement: Supplementary file 1 — Supplementary table. [file jcav13p0153s1.pdf]

Supplementary Table 1

Clinicopathologic Features of HCC Patients (n=20)

Gender	Male	15
	Female	5
Age	≤ 50	7
	> 50	13
AFP (μg/L)	≤ 20	4
	> 20	16
HBV	Positive	11
	Negative	9
Tumor size(cm)	≤ 5	6
	> 5	14
Capsular invasion	Yes	14
	No	6
Intrahepatic metastasis	Yes	9
	No	11
Distant metastasis	Yes	7
	No	13
Pathological differentiation	I-II	8
	III-IV	12
